# Toward High Throughput Core-CBCM CMOS Capacitive Sensors for Life Science Applications: A Novel Current-Mode for High Dynamic Range Circuitry

**DOI:** 10.3390/s18103370

**Published:** 2018-10-09

**Authors:** Saghi Forouhi, Rasoul Dehghani, Ebrahim Ghafar-Zadeh

**Affiliations:** 1 Department of Electrical and Computer Engineering, Isfahan University of Technology, 84156-83111 Isfahan, Iran; s.forouhi@ec.iut.ac.ir (S.F.); dehghani@cc.iut.ac.ir (R.D.); 2 Biologically Inspired Sensors and Actuators (BioSA), Department of Electrical Engineering and Computer Science (EECS), Lassonde School of Engineering, York University, Toronto, ON M3J 1P3, Canada

**Keywords:** CMOS, capacitive sensor, CBCM, dynamic range, bioengineering

## Abstract

This paper proposes a novel charge-based Complementary Metal Oxide Semiconductor (CMOS) capacitive sensor for life science applications. Charge-based capacitance measurement (CBCM) has significantly attracted the attention of researchers for the design and implementation of high-precision CMOS capacitive biosensors. A conventional core-CBCM capacitive sensor consists of a capacitance-to-voltage converter (CVC), followed by a voltage-to-digital converter. In spite of their high accuracy and low complexity, their input dynamic range (IDR) limits the advantages of core-CBCM capacitive sensors for most biological applications, including cellular monitoring. In this paper, after a brief review of core-CBCM capacitive sensors, we address this challenge by proposing a new current-mode core-CBCM design. In this design, we combine CBCM and current-controlled oscillator (CCO) structures to improve the IDR of the capacitive readout circuit. Using a 0.18 μm CMOS process, we demonstrate and discuss the Cadence simulation results to demonstrate the high performance of the proposed circuitry. Based on these results, the proposed circuit offers an IDR ranging from 873 aF to 70 fF with a resolution of about 10 aF. This CMOS capacitive sensor with such a wide IDR can be employed for monitoring cellular and molecular activities that are suitable for biological research and clinical purposes.

## 1. Introduction

Capacitive sensors have been receiving much attention due to their high resolution, low complexity, and low temperature-dependency. Among competing sensing technologies, CMOS, by offering a distinct cost and highly integrated circuits, offers great advantages for the development of capacitive sensors. These advantages include higher sensitivity, rapid detection, and the integration of electrical readout circuits and sensing electrodes on a single chip. In spite of microelectromechanical systems (MEMS)-based capacitive sensors such as accelerometers [1,2], position sensors [3,4], pressure sensors [5,6,7,8], and moisture sensors [9], a growing body of literature has studied capacitive sensors for Laboratory on a Chip (LoC) applications. These applications include DNA hybridization detection [10], protein interactions quantification [11], cellular monitoring [12,13,14], bio-particle detection [15], microRNA detection [16], organic solvent monitoring [17], sensing of droplet parameters [18], and bacteria detection [19,20].

**Figure 1:**
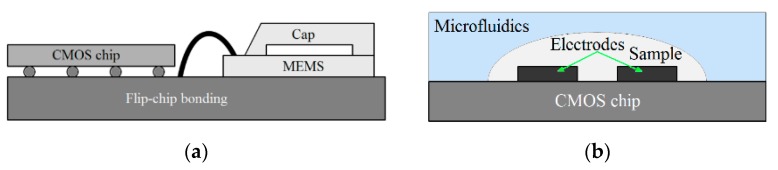
(**a**) Conceptual view of wire-bonded electrodes used for microelectromechanical systems (MEMS) applications, (**b**) conceptual view of on-chip electrodes used for Laboratory on a Chip (LoC) applications.

A capacitive sensor consists of sensing electrodes that are connected to an interface readout circuit, as shown in Figure 1a,b for both MEMS-based and LoC applications, respectively. The capacitive electrodes convert the physical [3,4], biological [10,11,12], and/or chemical parameters [21,22] in proximity of the electrodes into electrical signals. As illustrated in Figure 1a, the capacitive electrodes in MEMS-based applications such as accelerometers are used as an off-chip device wire-bonded (or flip-chip bonded) to the CMOS chip. For LoC applications, the sensing electrodes are realized above CMOS chip (see Figure 1b). The capacitive interface circuits are used to convert these signals into voltage, frequency, or other digital data [10,20,23,24]. A readout interface is required to accurately detect and process the sensed signals by electrodes. In CMOS-based lab-on-chip applications, microfluidic structures are implemented above CMOS sensing chips for steering biochemical samples through sensing electrodes. In these structures, various materials such as silicon, glass, and polymers can be formed using photolithography techniques [25,26,27]. Among them, polydimethylsiloxane (PDMS), SU-8, and polymethyl methacrylate are widely used for the development of low-cost disposable microfluidic structures. In addition to conventional high precision lithography, other techniques can also be utilized to fabricate microfluidic structures. These techniques include hot embossing [28], microinjection molding [29], soft lithography [30], photo-ablation [31], LIGA [32], 3D printing [33], and direct-write fabrication process (DWFP) [34]. In the development of hybrid microfluidic-CMOS systems, the biocompatibility and the reliability of the microfluidic packaging will be considered as key challenging issues. These challenges have been addressed by several researchers [30,34,35,36,37,38,39,40,41,42]. 

There is a considerable amount of literature on CMOS capacitive sensors for different LoC applications [12,15,20,23,24,43,44] using various circuitry methods. These methods include charge redistribution method (ChR), capacitance-to-frequency conversion (C2F), lock-in detection, and charge-based capacitance measurement (CBCM). 

Charge redistribution methods (ChR): Capacitive sensors may work based on charge redistribution principles using charge sharing (ChS) approaches [13,20], charge sensitive amplifiers (CSA) [45,46], or switched capacitor (SC) circuits [15,24,47]. In the charge sharing method, the voltage of a capacitor with known capacitance is proportional to the sensing capacitor whose charge is redistributed to the known capacitor using proper switches. The charge sharing circuit proposed in [20] occupies a small area, about 0.1 mm^2^ for a 16 × 16 array of sensor, but it has a limited sensitivity in comparison to the other sensors. In the charge sensitive amplifier-based methods, the readout circuit consists of a sensing and a reference capacitor, and an integrator, including an operational amplifier and an integrating feedback capacitor. A switch is also used to reset the voltage of the feedback capacitor. The output voltage of the amplifier is representative of the difference between the reference and sensing capacitances. A high resolution can be achieved using a charge sensitive amplifier as in [15] (about 21 aF for this example). In contrast to charge-sensitive amplifiers, which are controlled by voltage pulses, switched capacitor circuits use switches for charging and discharging capacitors. These circuits suffer offset, charge injection, and switching problems. Techniques such as double sampling and auto-zeroing can be used to mitigate these effects. The advantage of switched capacitor circuits is that they can be easily adapted to different analog-to-digital converters (ADCs) [24,48,49]. The sensing capacitance can be converted to digital by adding an ADC after switched capacitor-based CVCs, or by using switched capacitors inside the structure of different ADCs.

Capacitance-to-frequency conversion (C2F): This type of capacitive sensors converts sensing capacitances to frequency or period. These sensors utilize the structure of relaxation oscillators (RelO) [50,51] or ring oscillators (RO) [23,44,52,53,54,55]. Parasitic capacitances and temperature and process-dependent parameters of relaxation oscillators can affect the performance of the sensor. Ring oscillators are also sensitive to device sizes, temperature, supply voltage, and process. The advantage of the capacitance-to-frequency converters is that they produce semi-digital outputs. These sensors, like the ones reported in [23] and [44], demonstrate high resolutions in the range of tens of atto-Farad units (aF) (e.g., 21 aF, [14]). 

Lock-in detection: Among different techniques, lock-in detection is the highest resolution method that offers sub atto-Farad accuracy at the expense of more complexity and lower input dynamic range (IDR) (e.g., IDR = ~1 fF, [43]). In this method, the sensing signal, along with low frequency noise and offset, are modulated to high frequency. Then, the amplified sensing signal is recaptured by synchronous demodulation [43,56,57]. 

**Figure 2:**
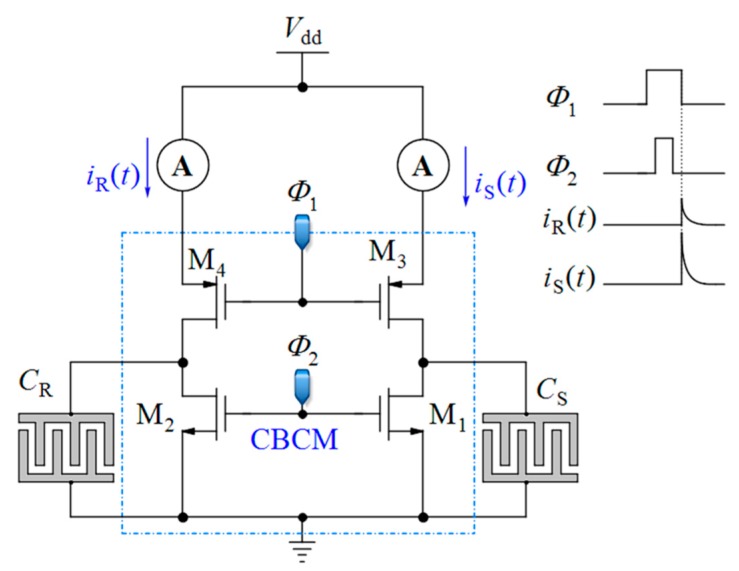
Core of the charge-based capacitance measurement (CBCM).

Charge-based capacitance measurement (CBCM): Another approach is the so-called charge-based capacitance measurement (CBCM) [58,59,60] (see Figure 2). This is a sensitive differential technique whose output current average is proportional to the difference between a sensing and a reference capacitance. Among the various CMOS-based techniques, the CBCM method has shown high accuracy along with the advantage of lower complexity for high throughput LoC applications. Despite these advantages, the previously reported core-CBCM capacitive sensors suffer from limited IDR. For instance, the core-CBCM capacitive sensor reported in [12] has an IDR about 10 fF in 0.35 μm CMOS technology. This problem arises from the intrinsic sharp exponential current of the core-CBCM (see *i*_S_(*t*) and *i*_R_ (*t*) in Figure 2). 

To the best of our knowledge, all of the previously reported core-CBCM capacitive sensors integrate the whole exponential output current of the core-CBCM circuit by an integrating capacitor, and then convert the integrating capacitor voltage to digital or frequency. Conversion of the whole exponential current to voltage and working in voltage mode limit the IDR of the capacitive sensor. This IDR problem becomes more serious for CMOS technologies with lower supply voltages, in particular for life science applications. 

In this paper, we focus on a new design to enhance the IDR of sensor using a current-mode method. In order to achieve higher than 10 fF IDR for cell analysis, current-mode techniques by using internal CMOS capacitors help to precisely follow the intrinsic sharp variations of the currents of core-CBCM circuits, in order to digitize and integrate them. In contrast to previous works [12,61,62,63,64,65] that average the whole CBCM current in the analog domain, the presented core-CBCM capacitive sensor does the required averaging in two steps, in both the analog and the digital domain. In this technique, a current-controlled oscillator (CCO) is used to frequency modulate the sharp exponential current of the CBCM circuit, and an adapted counter completes the integration in the digital domain. We believe our proposed solution advances previous core-CBCM methods by increasing its IDR. Additionally, in comparison to previous core-CBCM capacitance-to-frequency converters [66,67], digitization of the exponential CBCM current to very small units, and piecewise integration of this current helps to achieve a better resolution, and it is no longer necessary to use very large integrating capacitors to obtain high accuracy. Furthermore, digital outputs of capacitive sensors make reading them much easier, especially if they need to be able to connect to a microcontroller. Since the low-cost microcontrollers do not have internal ADCs, only digital [12,48,49] or semi-digital signals such as frequency modulated ones [23,67] are suitable as their inputs. 

The output pulses of the proposed core-CBCM capacitance-to-frequency converter have high frequencies, and they can be destroyed by the capacitance loading effects of the output pads. Thus, a Fibonacci reverse/forward linear feedback shift register (LFSR) is adapted as an on-chip up/down counter and a serial-in–serial-out register to count and register the number of pulses and also calibrate the offset current. 

In the remainder of this paper, Section 2 explains the basic principle of the CBCM method and gives a brief overview of related works on CMOS core-CBCM capacitive sensors. A new topology is proposed in Section 3. Additionally, an adapted Fibonacci reverse/forward LFSR is presented in this section. The related controlling clock strategies to meet the requirements of the sensor are described in Section 4. The simulation results will be demonstrated in Section 5, followed by a discussion in Section 6. Our conclusions are drawn in Section 7. 

## 2. Related Works

Core-CBCM capacitive sensors have been widely used for several LoC applications such as cell viability and proliferation monitoring [12,64], bio-particle sensing [68], organic solvent monitoring [17] and DNA detection [69]. As aforementioned, this method, by offering high accuracy and low complexity, has drawn considerable attention for high throughput screening in array structures. 

### 2.1. Principle of Core-CBCM Method 

The CBCM method was originally proposed for measurement of the crosstalk capacitances in between the conductors in deep CMOS chip [70,71]. A combination of this method with other circuitries was proposed for the detection of capacitive changes in the proximity of electrodes created on the topmost metal layer [58,59,60]. The basic core of the CBCM method is depicted in Figure 2. It is composed of a sensing (*C*_S_) and a reference capacitor (*C*_R_) that can be charged or discharged via two pairs of N-Channel CMOS (NMOS) (M_1_ and M_2_) and P-Channel CMOS (PMOS) (M_3_ and M_4_) switches controlled by clock pulses *Φ*_2_ and *Φ*_1_. These two clock pulses should be non-overlapped to avoid a short-circuit current. 

When *Φ*_1_ and *Φ*_2_ are both low, the capacitors *C*_S_ and *C*_R_ are charged via M_3_ and M_4_. When these pulses became high, the capacitors are discharged via M_1_ and M_2_. The instantaneous current of each branch, *i*_S_(*t*) and *i*_R_(*t*), can be obtained by:

(1) $$i_{S}\left(t\right)=C_{S}\frac{dv_{s}\left(t\right)}{dt}\;,$$

(2) $$i_{R}\left(t\right)=C_{R}\frac{dv_{R}\left(t\right)}{dt}\;,$$

where *v*_S_(*t*) and *v*_R_(*t*) are the instantaneous voltages across *C*_S_ and *C*_R_, respectively. The average of *i*_S_(*t*) and *i*_R_(*t*) over one period of *Φ*_1_ and *Φ*_2_ are obtained as follows:

$$\;I_{S}=\frac{1}{T_{s}}\int_{0}^{T_{s}}C_{S}\frac{dv_{s}\left(t\right)}{dt}dt\;$$

$$\;=\frac{C_{S}}{T_{s}}\int_{0}^{V_{\mathrm{dd}}-V_{\mathrm{Am}}}dv_{s}\;$$

(3) $$=\frac{C_{S}(V_{dd}-V_{\mathrm{Am}})}{T_{s}}\;,$$

$$\;I_{R}=\frac{1}{T_{s}}\int_{0}^{T_{s}}C_{R}\frac{dv_{R}\left(t\right)}{dt}dt\;$$

$$\;=\frac{C_{R}}{T_{s}}\int_{0}^{V_{\mathrm{dd}}-V_{\mathrm{Am}}}dv_{s}\;$$

(4) $$=\frac{C_{R}(V_{\mathrm{dd}}-V_{\mathrm{Am}})}{T_{s}}\;,$$

where *I*_S_ and *I*_R_ are the average of currents *i*_S_(*t*) and *i*_R_(*t*), respectively. *T*_S_ denotes the period of *Φ*_1_ and *Φ*_2_ and *V*_Am_ identifies the drop voltage over the ammeters. The average currents of two branches of this circuit, *I*_S_ and *I*_R_, are proportional to the related capacitances. Thus, the subtraction of *C*_S_ and *C*_R_ (Δ*C* = *C*_S_ − *C*_R_) can be obtained by the subtraction of these two averaged currents:

(5) $$I_{S}-I_{R}=\frac{\left(V_{\mathrm{dd}}-V_{\mathrm{Am}}\right).\left(C_{S}-C_{R}\right)}{T_{S}}\;,$$

In this way, Δ*C* can be extracted from *C*_S_ with a high accuracy. As a result, this method is suitable for LoC applications in which the sensing capacitances are very small. 

### 2.2. Single-Ended Core-CBCM Methods

**Figure 3:**
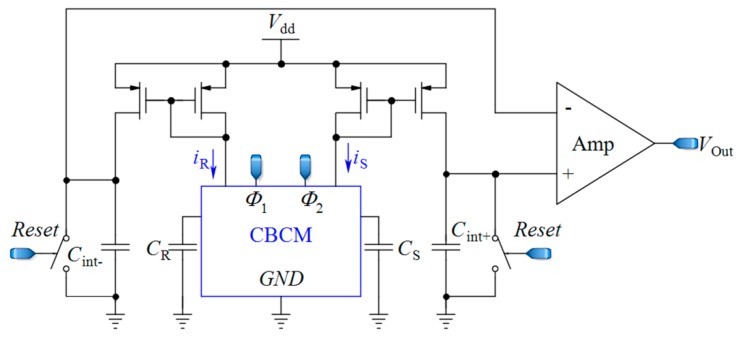
A core-CBCM capacitance-to-voltage converter (CVC) that integrates the currents *i*_S_ and *i*_R_ before subtraction (adapted from [61]).

To date, many efforts have been made to leverage the advantages of the CBCM method for life science applications [12,60,62] by proposing efficient circuit topologies. In these topologies, a sharp exponential current generated in the CBCM structure should be converted into digital structure. These topologies are designed to offer fast response, high sensitivity, high linearity, and high signal-to-noise ratios (SNRs) by reducing the external noises and parasitic capacitors. Evans et al. [61] made an attempt to develop the CBCM method by presenting an on-chip readout circuit, as shown in Figure 3. In this circuit, the currents *i*_S_ and *i*_R_ are separately averaged by two integrating capacitors, and then the resultant voltages are fed to a differential amplifier where those voltages are amplified and subtracted. In this approach, to achieve a higher sensitivity, high voltages across integrating capacitors are required. However, this situation pushes the differential amplifier to a nonlinear region and thus limits the resolution and the IDR of the sensor. In another effort, Stagni et al. [69] utilized the CBCM method for DNA recognition using off-chip readout circuitry. 

**Figure 4:**
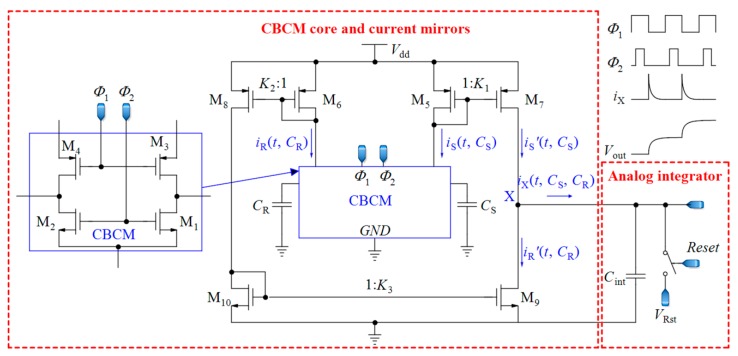
A core-CBCM CVC that subtracts the currents *i*_S_ and *i*_R_ before integration (adapted from [58]).

The first step toward a fully integrated core-CBCM capacitive sensor was taken by Ghafar-Zadeh et al. [58] by proposing a new single-ended CVC circuit that was interconnected to microelectrodes. In this sensor, the microelectrodes were implemented on the topmost layer of a CMOS technology and used as sensing electrodes for LoC applications. To mitigate the IDR problem of the approach used by Evans et al. [61], Ghafar-Zadeh et al. [58] proposed a new circuit to subtract the currents *i*_S_ and *i*_R_ before injection to the integrating capacitor, as shown in Figure 4. In the first block of Figure 4, the currents of CBCM block, *i*_S_ and *i*_R_, are mirrored, amplified, and then subtracted by using the current mirrors realized by M_5-10_. It is straightforward to verify that the instantaneous current *i*_X_(*t*, *C*_S_, *C*_R_) in Figure 4 is equal to Equation (6):

$$\;i_{X}\left(t,\;C_{S},C_{R}\right)=i_{S}^{'}\left(t,\;C_{S}\right)-i_{R}^{'}\left(t,\;C_{R}\right)\;$$

(6) $$\;=K_{1}i_{S}\left(t,\;C_{S}\right)-K_{2}K_{3}i_{R}\left(t,\;C_{R}\right)\;$$

$$=K_{1}C_{S}\frac{dv}{dt}-K_{2}K_{3}C_{R}\frac{dv}{dt}\;,$$

where *K*_1_, *K*_2_ and *K*_3_ stand for the gain of the first (M_5,7_), the second (M_6,8_), and the third (M_10,9_) current mirrors, respectively. To achieve a symmetric structure, it is better to choose *K*_3_ = 1 and *K*_1_ = *K*_2_. If *K*_1_ = *K*_2_ = *K*, the integration of *i*_X_ during one period of *Φ*_1_ and *Φ*_2_ can be expressed as follows:

(7) $$\int_{0}^{T_{S}}i_{X}\left(t,\;C_{S},C_{R}\right)dt\approx K\left(V_{\mathrm{dd}}-V_{\mathrm{thp}}\right)\Delta C\;,$$

where *V*_dd_ is the power supply voltage and *V*_thp_ is the PMOS threshold voltage. Sensing and reference capacitors are charged to nearly *V*_dd_-*V*_thp_ during the charging interval. Averaging over *N* cycles of *Φ*_1_ and *Φ*_2_, we can improve the sensitivity of the sensor. Let us assume that the total integration time is *T*_int_ = *NT*_S_, and we obtain the integration of *i*_X_ during this interval as follows:

(8) $$\int_{0}^{T_{\mathrm{int}}}i_{X}\left(t,\;C_{S},C_{R}\right)dt=NK\left(V_{\mathrm{dd}}-V_{\mathrm{thp}}\right)\Delta C\;,$$

which is proportional to Δ*C*. In [58], the current *i*_X_ is integrated in the analog domain by the second block shown in Figure 4 using an integrating capacitor. As the continuation of that work, a single-ended sigma delta (ΣΔ) modulator was designed for converting the analog voltage to digital data [17]. Contrary to conventional ADCs, the input analog voltage of this ΣΔ modulator is a step signal instead of a ramp. In other efforts, they showed the ability of their proposed circuit to monitor the organic solvent and bacteria growth [62,72]. In [73], the CBCM technique is used in a 256 × 256 array for high impedance spectroscopy and imaging where the CBCM currents are integrated by integrating capacitors, and the output voltages are digitized by eight ADCs.

### 2.3. Fully Differential Core-CBCM Capacitive Sensor Method

**Figure 5:**
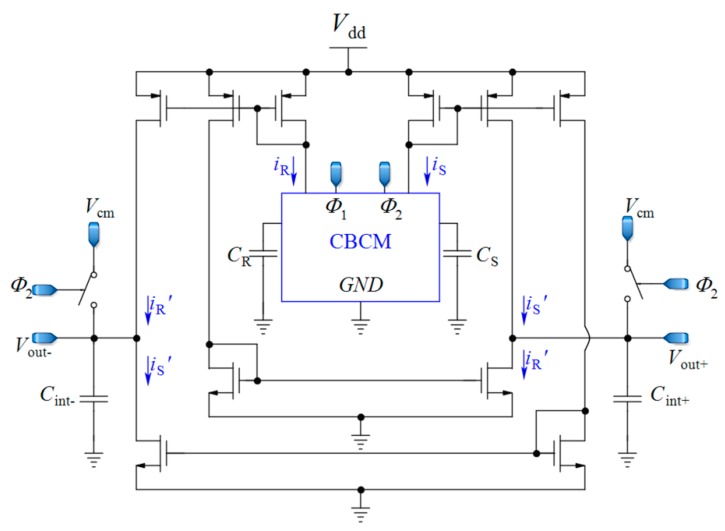
Fully differential core-CBCM CVC adapted from [60,64].

As the continuation of the work discussed in the last Section 2.2, a fully differential core-CBCM CVC was proposed by Prakash et al. in order to double the IDR and to mitigate the effect of common mode noise and the parasitic capacitances of these single-ended core-CBCM capacitive sensors, for cell-sensing applications [60,64] (Figure 5). In [74], cascade current mirrors were used in the same structure to improve the output impedance of current mirrors and to achieve better results for impedance measurement. In another effort, a fully differential core-CBCM capacitance-to-digital converter was reported in [65] by using an adapted fully differential sigma delta (ΣΔ) modulator. This modulator can be used to convert the capacitance to digital in an array of core-CBCM capacitive sensor [12]. Although the integration of the currents *i*_S_ and *i*_R_ after subtraction in both single-ended [17,58,62,72] and fully differential architectures [12,60,64,65] shows wider IDRs in comparison to the approach depicted in Figure 3 [61], averaging the whole amplified differential current of the CBCM core by integrating capacitors still limits the IDR. 

### 2.4. Core-CBCM Capacitance-to-Frequency Converter

**Figure 6:**
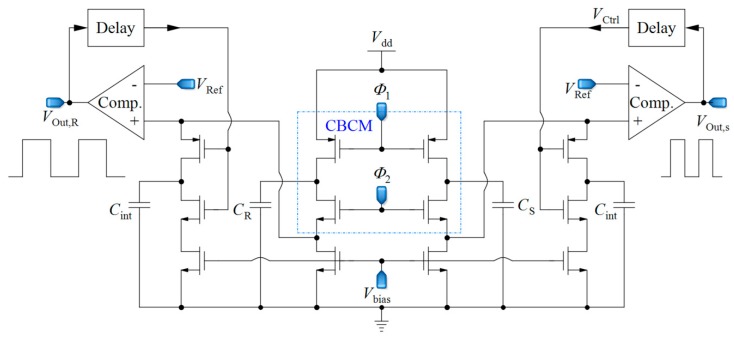
A core-CBCM capacitance-to-frequency converter that integrates all of the exponential CBCM currents in the analog domain and converts them to two frequencies (adapted from [66]).

In [66], a capacitive sensor is proposed, utilizing a combination of CBCM method and capacitance-to-frequency conversion, as illustrated in Figure 6. In this circuit, the sharp exponential currents of each branch of the CBCM block are averaged using a large integrating capacitor (*C*_int_) in the analog domain, and then this voltage is converted to frequency by a comparator. Two similar structures are used for each of the sensing and reference capacitors in order to convert the related capacitance to frequency. The subtraction of the output frequencies of the two branches is proportional to Δ*C*. In this circuit, the whole integration of the CBCM exponential current is performed in the analog domain, as with the previous circuits and then the resultant voltage is converted to frequency. This circuit requires a very large integrating capacitor to achieve a proper charging time difference, and it works from a femto-Farad to pico-Farad range, which is not suitable for LoC applications. Yamane et al. [67] used a similar approach by an integrator and a Schmitt trigger. However, it also suffers from low resolution.

In sum, all aforementioned core-CBCM capacitive sensors reported in the literature work in a voltage mode and integrate the whole exponential CBCM current in the analog domain using integrating capacitors that convert the current to voltage. As a result, the IDR of the sensing capacitance is limited by the voltage swing of the integrating capacitor. Current-mode circuits are more suitable for low supply voltage CMOS technologies. Thus, this paper proposes a novel core-CBCM capacitance-to-frequency converter working in current-mode in order to increase IDR. Utilizing the internal capacitors of CMOS transistors makes it possible to follow the sharp variations of CBCM currents and to use both the analog and the digital domains to integrate CBCM currents.

## 3. Proposed Core-CBCM Capacitive Sensor

**Figure 7:**
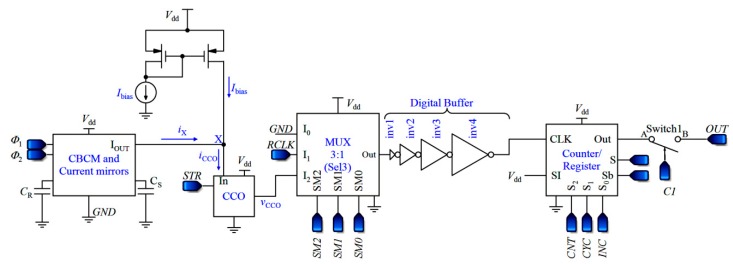
Block diagram of the proposed capacitive sensor.

In the proposed capacitive sensor shown in Figure 7, the currents of the two branches of the CBCM core are amplified and subtracted using the current mirrors and then fed into a CCO. The CCO should be able to follow the variations of the sharp exponential currents of the CBCM block, so that it modulates them to pulse frequency. Since the average of the differential current is proportional to Δ*C*, a counter is used to average the output frequency. In other words, unlike previous works, in the presented sensor, the required averaging operation is not done only in the analog domain. More detailed descriptions of the proposed capacitive sensor are presented in the following subsections. 

### 3.1. Description of the Sensor Performance 

Prior to describing the sensor performance let us calculate the integration of the input current of the CCO block over a specific time. Thereafter, two approaches are put forward to achieve a practical result that is proportional to Δ*C*.

#### 3.1.1. Core-CBCM Block Performance and the Bias Current

The analog circuit of the first block of Figure 7 is the first block of the circuit illustrated in Figure 4, including the CBCM core and current mirrors (without the analog integrator). A direct current, *I*_bias_, is also added to *i*_X_ to shift the CCO operating area to its linear region (*i*_CCO_ = *i*_X_ + *I*_bias_). Assuming *T*_int_ = *NT*_S_, we can obtain the integration of *i*_CCO_ during this interval as follows:

(9) $$\int_{0}^{T_{\mathrm{int}}}i_{\mathrm{CCO}}\left(t,\;C_{S},C_{R}\right)dt=NK\left(V_{\mathrm{dd}}-V_{\mathrm{thp}}\right)\Delta C+I_{\mathrm{bias}}T_{\mathrm{int}}\;,$$

which changes with Δ*C* linearly.

#### 3.1.2. The First Description of the Sensor Performance

**Figure 8:**
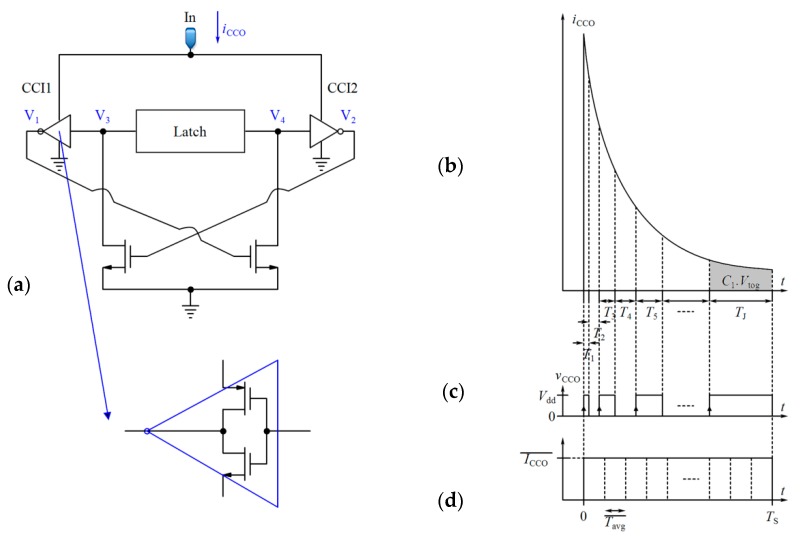
(**a**) Basic design of the CCO [75], (**b**) CCO input current, (**c**) CCO output voltage, (**d**) CCO averaged input current based on the mean value theorem.

The basic design of a CCO from [75] is illustrated in Figure 8a. In this circuit, the nodes V_1_ and V_2_ are alternatively charged by the input current of the CCO (*i*_CCO_), and they produce the output voltages V_3_ and V_4_. Because of the latch, V_3_ and V_4_ are the inversions of each other. The frequency of the output nodes depends on the time that is required for V_1_ and V_2_ to reach a toggling voltage (*V*_tog_) in order to change the state of the latch. Thus, the integration of *i*_CCO_ in the required interval (*T*_m_) for V_1_ (or V_2_) to reach *V*_tog_ is obtained as follows:

(10) $$\int_{\left(\sum_{z=1}^{m}T_{z}\right)-T_{m}}^{\sum_{z=1}^{m}T_{z}}i_{\mathrm{CCO}}\left(t,\;C_{S},C_{R}\right)dt=C_{1}V_{\mathrm{tog}}\;,\;m=1,2,\ldots ,\;J\;,$$

where *C*_1_ is the total capacitance seen at nodes V_1_ or V_2_ (for a symmetrical CCO circuit). *C*_1_ consists of the junction capacitances of the transistors, and is very small. So, a little change in *i*_CCO_ results in a significant variation in the CCO output frequency. As *C*_1_*V*_tog_ is almost constant, the larger amplitude of *i*_CCO_ leads to shorter interval (*T*_m_) and vice versa. Thus, as shown in Figure 8b, the total area under *i*_CCO_(*t*) during a specified time can be divided into very small blocks with an area of *C*_1_*V*_tog_ for each one (Here we call them unit blocks). For example, *J* unit blocks with the area of *C*_1_*V*_tog_ can be obtained during *T*_S_ (Figure 8b–d shows this concept by exaggeration in the sizes of the unit blocks). After each *T*_m_ (*m* = 1, 2,…, J), the state of the output voltage of the CCO (*v*_CCO_) changes from low to high or conversely high to low (Figure 8c). The integration of *i*_CCO_ during *T*_int_ is equal to Equation (11):

$$\;\int_{0}^{T_{\mathrm{int}}}i_{\mathrm{CCO}}\left(t,\;C_{S},C_{R}\right)dt=N\overset{J}{\underset{m=1}{\sum }}\left(\int_{\left(\sum_{z=1}^{m}T_{z}\right)-T_{m}}^{\sum_{z=1}^{m}T_{z}}i_{\mathrm{CCO}}\left(t,\;C_{S},C_{R}\right)dt\right)\;$$

(11) $$=NJC_{1}V_{\mathrm{tog}}\;,$$

A counter can be used to count these blocks. In this way, the integration process will be completed in the digital domain. If the counter counts only the rising edges of the pulses, the number counted by the counter (*Numb*) during *T*_int_ will be equal to *NJ*/2. It is easy to verify that using Equation (12):

(12) $$Numb=\frac{NK\left(V_{dd}-V_{\mathrm{thp}}\right)\Delta C}{2C_{1}V_{\mathrm{tog}}}\;,$$

where *N* is an even number. Assuming *V*_tog_ is constant, the relation between *Numb* and Δ*C* will be linear. 

#### 3.1.3. The Second Description of the Sensor Performance

**Figure 9:**
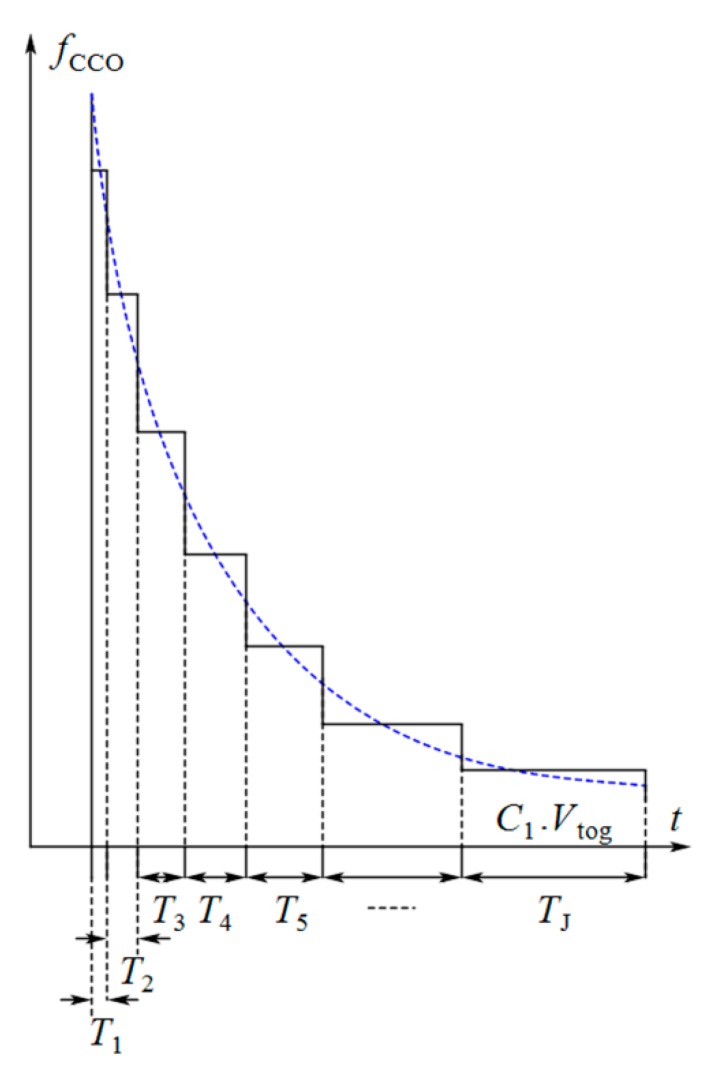
Frequency of the output pulses of the CCO and its envelope (by exaggeration in the sizes of the unit blocks).

One may argue that the sharp exponential input current of the CCO is a frequency modulated current. Although the CCO does not convert its input current to frequency in every moment of the integration time, its output frequency follows the envelope of the discrete variable frequency of the output pulses (see Figure 9 as an exaggerated illustration). In an ideal case, the output frequency of the CCO is given by Equation (13):

(13) $$f_{\mathrm{CCO}}\left(t,\;C_{S},C_{R}\right)=M.i_{\mathrm{CCO}}\left(t,\;C_{S},C_{R}\right)\;,$$

where *M* denotes the gain of the CCO. Combining Equations (6) and (13) and averaging the result over one period of *Φ*_1_ and *Φ*_2_, we deduce the following equation:

(14) $$F_{\mathrm{CCO}}\left(\Delta C\right)=\frac{M.K.\Delta C.\left(V_{\mathrm{dd}}-V_{\mathrm{thp}}\right)}{T_{S}}+M.I_{\mathrm{bias}}\;,$$

where *F*_CCO_ identifies the average of the variable frequencies of the output pulses over *T*_S_ and *V*_thp_ is the threshold voltage of the PMOS transistors. For the total averaging time (*T*_int_), it can be simply shown that:

(15) $$F_{\mathrm{CCO}}\left(\Delta C\right).T_{\mathrm{int}}=M.N.K.\Delta C.\left(V_{\mathrm{dd}}-V_{\mathrm{thp}}\right)+M.I_{\mathrm{bias}}.T_{\mathrm{int}}\;,$$

As seen from Figure 8c, a half period of each pulse is produced for each unit block. We can therefore use the mean value theorem to obtain Equation (16):

(16) $$\int_{0}^{T_{\mathrm{int}}}f_{\mathrm{CCO}}\left(t,\;C_{S},C_{R}\right).dt=F_{\mathrm{CCO}}\left(\Delta C\right).T_{\mathrm{int}}=\frac{NJ}{2}\;,$$

which is equal to the counter number (*Numb*). Thus, the sensitivity of the sensor can be obtained by Equation (17):

(17) $$S_{\frac{d\left(Numb\right)}{d\left(\Delta C\right)}}=M.N.K.\left(V_{\mathrm{dd}}-V_{\mathrm{thp}}\right)\;,$$

Equation (17) shows that there are three parameters affecting the sensitivity of the sensor including the gain of the current mirrors used in the first block (*K*), the sensitivity of the CCO (*M*), and the number of the cycles of *Φ*_1_ and *Φ*_2_ (*N*), with the latter being off-chip controllable. It should be pointed out that the IDR of the CCO and the size of the counter can limit the IDR of the sensor. A trade-off should be considered for the value of *K*. The more gain that is dedicated to the first block, the less IDR that is achieved for the sensor. However, this results in greater sensitivity. The sensitivity can be increased by two other parameters, *N* and *M*. However, rising *N* brings about a longer measurement time.

In summary, the proposed circuit integrates the current signals in each period in two steps. First, the CCO integrates the current over the time required for each unit block in the analog domain. Then, the counter counts the unit blocks and completes the integration in the digital domain. Each unit block produces an individual frequency at the output of the CCO. Finally, a string of the pulses with discrete frequencies will appear at the output of the CCO that follows the envelope of the instantaneous frequency modulated current response of the core-CBCM circuit (Figure 9). The small junction capacitances of the transistors used as the integrating capacitor of the CCO make it capable of high digitization of the sharp exponential current of the core-CBCM circuit. On the other hand, the current-mode operation of the CCO helps to overcome the IDR limitation that is imposed by the supply voltage.

### 3.2. Current-Controlled Oscillator (CCO)

#### 3.2.1. Performance of the Used CCO

**Figure 10:**
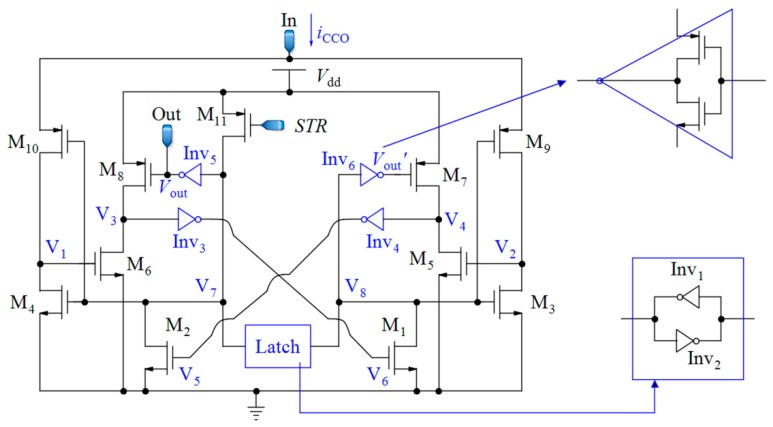
Complete current-controlled oscillator proposed in [75].

Since the waveform of the current *i*_CCO_(*t*) is very sharp, the CCO should be able to follow the fast variations in *i*_CCO_(*t*). Here, the CCO proposed in [75] was selected because of its striking features, such as wide IDR and very low sensitivity to power supply voltage variations (Figure 10).

For the performance description of this CCO, we can start from node V_7_. If the voltage of this node (V_7_) is high, M_10_ is turned off; while M_4_ is turned on, discharges the voltage at node V_1_ to zero. At the same time, the voltage of V_8_ is low and M_3_ is in the cut-off region. Additionally, M_9_ is in the saturation region and steers the input current *i*_CCO_ to charge the total capacitance that is seen at node V_2_. When the rising voltage of V_2_ reaches the threshold voltage of M_5_, this transistor is turned on and pulls down the voltage at node V_4_. When this voltage is about half of the supply voltage (*V*_dd_/2), through Inv_4_, M_2_ is turned on and it changes the state of the latch that is composed of the two inverters, Inv_1_ and Inv_2_. In the new state, the input current *i*_CCO_ charges the total capacitance at node V_1_, and the voltage of V_2_ becomes zero. An alternative performance of this process brings about the output pulses whose frequencies are proportional to the input current of CCO (*i*_CCO_). It can be proven that the relation between the frequency of the output pulses and the input current is obtained by Equation (18) [75]:

(18) $$f_{\mathrm{CCO}}=\frac{1}{T_{\mathrm{CCO}}}=\frac{i_{\mathrm{CCO}}}{nC_{1}V_{T}\ln\left(1+\frac{i_{\mathrm{CCO}}C_{3}V_{\mathrm{dd}}L_{6}}{2nC_{1}V_{T}I_{0}W_{6}}e^{\left(V_{\mathrm{thn}}/nV_{T}\right)}\right)}\;,$$

where *I*_0_ stands for a technology-dependent scaling parameter, *V*_T_ denotes the thermal voltage, and *n* identifies the subthreshold gate coupling coefficient. *C*_1_ and *C*_3_ are the total capacitances that are seen at nodes V_1_ and V_3_, respectively. *V*_thn_ is the NMOS threshold voltage, *W*_6_ denotes the channel width, and *L*_6_ is the channel length of M_6_. Both transistors M_5_ and M_6_, have the same sizes because of symmetry, and they operate in the sub-threshold region. According to Equation (18), and knowing that *C*_1_ is a very small capacitor; the output frequency is proportional to *i*_CCO_. The large sizes of M_5_ and M_6_ help to reduce the effects of variations in the process parameters and the mismatches on the capacitor *C*_3_, but at the expense of less sensitivity of the CCO. Thus, a trade-off should be considered to achieve a suitable linearity and sensitivity. The transistor M_11_ is used to start-up the oscillation controlled by signal *STR*. When *STR* is low, the voltage at node V_7_ rises up toward *V*_dd_, and after commencing the oscillation of CCO, M_11_ is turned off.

#### 3.2.2. Effect of the Nonlinearity of the CCO on the Sensor Response

Since the toggling voltage at nodes V_1_ or V_2_ (*V*_tog_) of the circuit shown in Figure 10 is dependent on the input current *i*_CCO_, the CCO has a nonlinear behavior. Equation (18) reveals that the logarithmic term in the denominator is the main reason for this nonlinearity. Although this CCO is designed for a direct current in [75], it can be proven that it is also useful for our time-varying current. Based on the mean value theorem, the area under the graphs of the signals shown in Figure 8b,d are the same. In Figure 8d, $\bar{I_{\mathrm{CCO}}}=\frac{1}{T_{S}}\int_{0}^{T_{S}}i_{\mathrm{CCO}}\left(t,\;C_{S},C_{R}\right)dt+I_{\mathrm{bias}}$ and $\bar{T_{\mathrm{avg}}}=\frac{T_{S}}{J}$. Thus, it is straightforward to verify that:

(19) $$\frac{1}{\bar{T_{\mathrm{avg}}}}=\frac{J}{T_{S}}=\frac{K\left(V_{\mathrm{dd}}-V_{\mathrm{thp}}\right)\Delta C/T_{S}+I_{\mathrm{bias}}}{nC_{1}V_{T}\ln\left(1+\frac{\left(K\left(V_{\mathrm{dd}}-V_{\mathrm{thp}}\right)\Delta C/T_{S}+I_{\mathrm{bias}}\right)C_{3}V_{\mathrm{dd}}L_{6}}{2nC_{1}V_{T}I_{0}W_{6}}e^{\left(V_{thn}/nV_{T}\right)}\right)},$$

Since the number of rising edges during *T*_int_ is equal to *NJ*/2, the relation between this number (*Numb*) and Δ*C* can be calculated by:

(20) $$Numb=\frac{NK\left(V_{\mathrm{dd}}-V_{\mathrm{thp}}\right)\Delta C+I_{\mathrm{bias}}T_{\mathrm{int}}}{2nC_{1}V_{T}\ln\left(1+\frac{\left(K\left(V_{\mathrm{dd}}-V_{\mathrm{thp}}\right)\Delta C+I_{\mathrm{bias}}T_{S}\right)C_{3}V_{\mathrm{dd}}L_{6}}{2nC_{1}V_{T}I_{0}W_{6}T_{S}}e^{\left(V_{\mathrm{thn}}/nV_{T}\right)}\right)},$$

Using the approximations of ln(1+x)≈x and $\frac{1}{1+x}\approx 1-x$ for |x|<<1, we can find a straightforward relation, as shown in Equation (21):

(21) $$Numb=a_{0}+a_{1}.\Delta C+a_{2}.\Delta C^{2}\;,$$

If$\;F≜\frac{I_{\mathrm{bias}}T_{S}C_{3}V_{\mathrm{dd}}L_{6}}{2nC_{1}V_{T}I_{0}W_{6}T_{S}}\exp\left(\frac{V_{\mathrm{thn}}}{nV_{T}}\right)$, the values of $a_{0}$, $a_{1}$, and $a_{2}$ can be obtained as follows:

(22) $$a_{0}\approx \frac{I_{\mathrm{bias}}T_{\mathrm{int}}}{2nC_{1}V_{T}ln\left(F\right)},$$

(23) $$a_{1}\approx \frac{NK\left(V_{\mathrm{dd}}-V_{\mathrm{thp}}\right)}{2nC_{1}V_{T}\ln\left(F\right)}\left(1-\frac{1}{2nC_{1}V_{T}\ln\left(F\right)T_{S}}\right),$$

(24) $$a_{2}\approx \frac{-NK^{2}{\left(V_{\mathrm{dd}}-V_{\mathrm{thp}}\right)}^{2}}{{\left(2nC_{1}V_{T}\right)}^{2}I_{\mathrm{bias}}T_{S}}\;,$$

where *F* >> 1 and $a_{0}\gg a_{1}\gg \left|a_{2}\right|$. Thus, there is second-order nonlinearity in the final response of the capacitive sensor which is reduced by increasing *I*_bias_.

### 3.3. Counter and Register

The employed counter should be fast enough to follow the pulses with variable frequencies. The Fibonacci linear feedback shift register (LFSR) is chosen due to its high speed, low circuit complexity, and thus its small area. Furthermore, it can be utilized as both a counter and a serial-input serial-output register. 

The presence of bio-particles or other remnants in the micro-channels and the non-idealities of the circuit such as parasitic capacitances, mismatches in the current mirrors, the effects of temperature variations cause an offset current for Δ*C* = 0. Furthermore, the bias current, *I*_bias_, and its non-idealities are added to the offset current. This offset current might saturate the system. Thus, there should be enough of a secure margin between the IDR of the CCO and the maximum mirrored current of the core-CBCM circuit. Here, the circuit is calibrated by designing an LFSR-based up/down counter. When the sensing electrode is not exposed to the biochemical sample (Δ*C* = 0), the shift register shifts the data reversely. Then, the resultant value is registered as the initial value of LFSR for up-counting. After applying the sample to the sensing electrode, the counter commences forward shifting of the registered initial data. In other words, this approach helps to omit the large term of $a_{0}$ from Equation (21), and thus, it results in higher measurement accuracy. 

**Figure 11:**
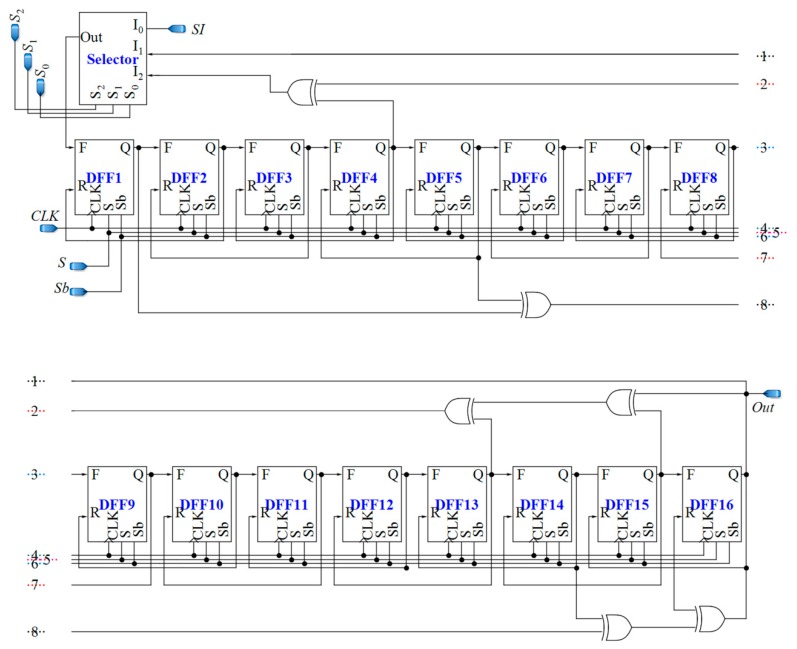
Proposed 16-bit LFSR-based reverse/forward counter/register.

The 16-bit counter used in the proposed sensor is a combined and adapted version of the bidirectional LFSR proposed in [76], and the LFSR-based counter used in [77]. The feedback polynomials of LFSR for forward counting are known. In order to find feedback polynomials for reverse counting, the Karnaugh map can be used. Since using a Karnaugh map for 16 bits is overwhelming, we will start with smaller sizes of LFSRs. As seen in Table 1, there is a repetitive relation between the structures of the reverse and forward LFSRs with the same sizes. Thus, it is possible to guess the feedback polynomial for a reverse LFSR, based on the polynomial for forward counting. The feedback polynomial for a 16-bit forward LFSR is x^16^ + x^15^ + x^13^ + x^4^ + 1, and based on the repetitive trend shown in Table 1, the one for reverse counting will be x^16^ + x^14^ + x^5^ + x^1^ + 1. The utilized LFSR-based reverse/forward counter/register is depicted in Figure 11.

**Figure 12:**
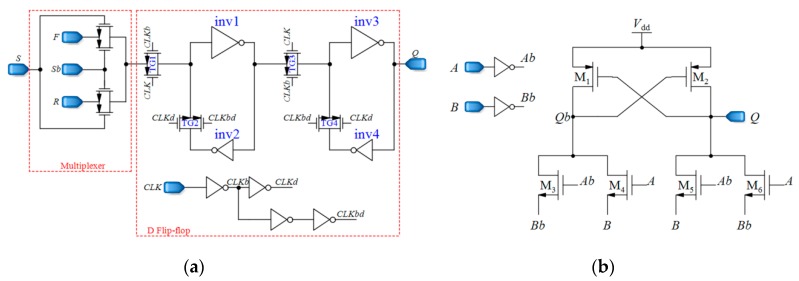
(**a**) Fast static D flip-flop, (**b**) Fast XOR gates based on a differential cascade voltage switch with a pass-gate (DCVSPG) [78].

D flip-flops used in the up/down counter should be fast and static. This is because the output pulses of the CCO have various frequencies, and the D flip-flops should be able to follow the pulses and save their own data until the next pulse comes. Additionally, two different inputs are required for D flip-flops, one for reverse shifting, and the other one for forward shifting. Figure 12a demonstrates the D flip-flop that is used in the counter, which is composed of two blocks, a 2:1 multiplexer and the fast static D flip-flop that is presented in [79]. The controlling signal *Sb* of the multiplexer is the inverted version of *S*. By using the multiplexer, the required input is selected and fed to the D flip-flop. *F* and *R* inputs are for forward and reverse counting, respectively. Moreover, the XOR gates used in the counter should be fast enough. Figure 12b shows the XOR gate proposed in [78], operating based on a differential cascade voltage switch with a pass-gate (DCVSPG). The multiplexers used in the sensor and their controlling signals make the sensor operate in five different phases, which is described in the next section. Also, a digital buffer is placed between the CCO and the counter to avoid changing the frequencies of the output pulses of the CCO due to the counter-loading effect (Figure 7).

### 3.4. Interdigitated Microelectrodes

**Figure 13:**
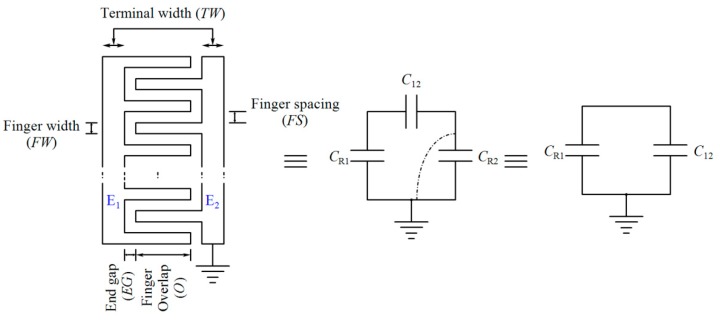
Model of interdigitated electrodes.

Sensing and reference capacitors are co-planar interdigitated electrodes that are fabricated by the topmost layer of CMOS technology. The parasitic capacitors of the electrodes are depicted in Figure 13. Each electrode consists of two parts, E_1_ and E_2_. *C*_R1_, *C*_R2_, and *C*_12_ are the parasitic capacitances between E_1_ and the substrate, between E_2_ and the substrate, and between E_1_ and E_2_, respectively. Since one side of the electrode, E_2_, is grounded, the total capacitance seen at the node of the reference electrode is equal to the parallel combination of *C*_R1_ and *C*_12_. For the sensing electrode, the corresponding capacitance of the aqueous sample which is applied to the electrode should be added to *C*_R_.

## 4. Clocking Strategy of the Sensor

**Figure 14:**
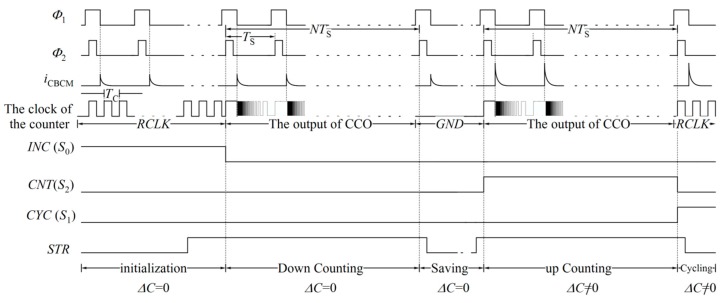
Different waveforms of the proposed sensor.

The proposed sensor works in five phases. The waveforms of the sensor signals and the controlling signals corresponding to each phase are depicted in Figure 14 and Table 2. These phases are explained as follows.

Initialization: Before employing the biochemical sample to the electrodes, by rising the signal *INC*, which is connected to the selecting pin S_0_ of the counter (as shown in Figure 7 and Figure 11), all D flip-flops receive the input *SI*, which is connected to the supply voltage. In this phase, D flip-flops work with an optional low frequency clock pulse (*RCLK*) with a period of *T*_C_. After 16*T*_C_, all D flip-flops are ready to start counting.

Down counting for Δ*C* = 0: In this phase, the sensing capacitor, *C*_S_, is free of biochemical sample. The output of the CCO, which has already started its oscillation, is fed to D flip-flops as their clock pulses. Simultaneously, the counter is set to count reversely. The counter should shift the data in the reverse direction over an interval that is equal to *T*_int_=*NT*_S_. In this situation, the signal *Sb* of D flip-flops is high, and all three signals *CYC*, *INC*, and *CNT* are low. Thus, the D flip-flops receive the data on their R input.

Saving mode (Δ*C* = 0): In this phase, the sensing capacitor is not yet exposed to the biochemical sample. However, the controlling signal, *SM0*, is high. Thus, the D flip-flops of the counter receive no pulses and they act as registers.

Up-counting (Δ*C* ≠ 0): In this phase, the biochemical sample is applied to the sensing capacitor and the counter is adjusted for forward counting of the output pulses of the CCO. The controlling signals *INC* and *CYC* are low, and the signal *CNT* is high for an interval equal to *T*_int_.

Cycling: The number of the counted pulses during *T*_int_ goes out serially at a particular frequency (1/*T*_C_). Thus, *RCLK* is fed to D flip-flops again, which has a low frequency equal to *f*_C_ = 1/*T*_C_. The low frequency of *RCLK* mitigates the capacitance loading effect of the output pin. 

## 5. Results

The proposed sensor is simulated in a 0.18 μm CMOS technology. The post-layout simulation results are shown in this section. The CCO is the core of the sensor that should be capable of following the demonstrated and discussed specifications. 

### 5.1. Linear Region of the CCO

**Figure 15:**
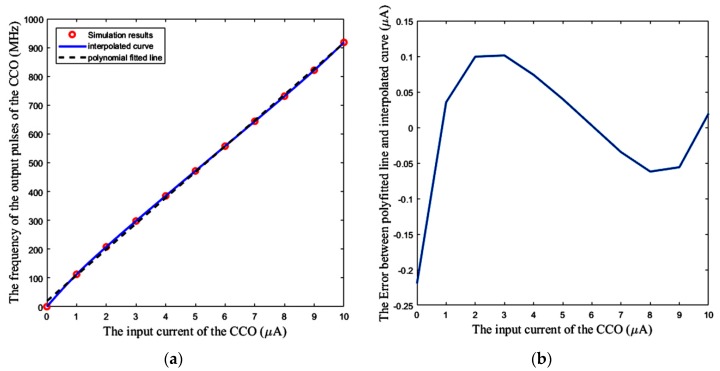
(**a**) CCO output frequency vs DC input current, (**b**) Error between the polynomial fitted line and the simulation results.

Figure 15a shows the response of the CCO separately, which is examined by applying a DC input current to the CCO, and the measurement of the frequency of its output pulses. The nonlinearity error for lower input currents is higher, as seen in Figure 15b. The added bias current *I*_bias_ shifts the operating area of the CCO to the more linear region, and thus, helps to have a CCO with more linear behavior. This is equivalent to decrease $a_{2}$ in Equation (21) for the whole sensor. 

### 5.2. The Response of the Analog Part of the Sensor 

**Figure 16:**
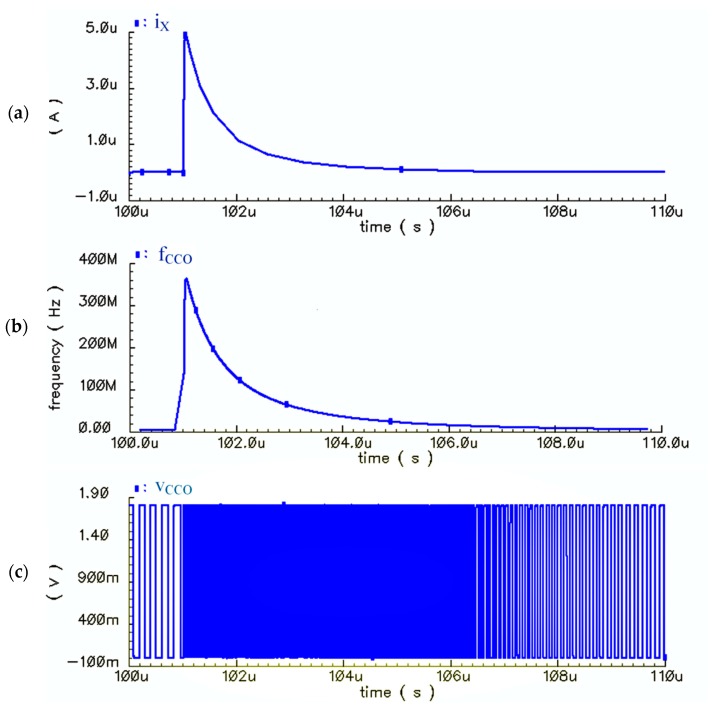
(**a**) Variations of the CBCM output current (*i*_X_) versus time, (**b**) Variations of the output frequency of the CCO (*f*_CCO_) versus time, (**c**) Variations of the CCO output voltage pulses (*v*_CCO_) for the CBCM output current as the CCO input (*i*_CCO_ = *i*_X_) for Δ*C* = 60 fF (in order to show the compact high frequency pulses more clearly and to lower their frequencies, *I*_bias_ is omitted from the input of the CCO).

Figure 16a–c shows the transient response of the proposed sensor for a specific Δ*C* (60 fF). The input current, the output frequency (*f*_CCO_), and the output voltage of the CCO (*v*_CCO_) are illustrated in Figure 16a–c, respectively. Since the frequencies of the output pulses of the CCO are so high, the pulses are so compact that they cannot be distinctly illustrated for just one period of *Φ*_1,2_ (*T*_S_). Thus, in Figure 16, the bias current is omitted (*i*_CCO_ = *i*_X_) to lower the frequency level of output pulses, and to show the concept of the proposed solution more clearly. Based on the waveforms shown in Figure 16, the CCO has enough speed for following the sharp exponential currents of the core-CBCM circuit. It is worthwhile noting that the jitter of the CCO does not affect the sensor response significantly. This is because the integration time (*T*_int_ = *NT*_S_) is long enough so that the jitter averages out at zero.

**Figure 17:**
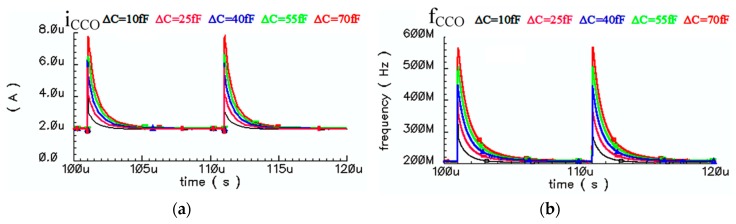
(**a**) Input current of the CCO, (**b**) CCO output frequency versus five different Δ*Cs*.

The input current of the CCO, considering the required bias current (*i*_CCO_ = *i*_X_ + *I*_bias_) and the output frequency of the CCO are depicted for five different Δ*C*s versus time in Figure 17a,b, respectively. 

**Figure 18:**
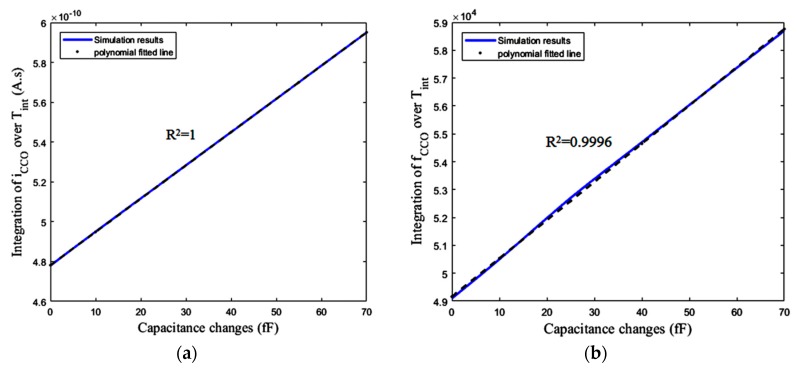
(**a**) Integration of *i*_CCO_ over *T*_int_ versus Δ*Cs*, (**b**) Integration of *f*_CCO_ over *T*_int_ versus Δ*Cs*.

Figure 18a,b shows the integration of *i*_CCO_ and *f*_CCO_ over the total integration time (*T*_int_) versus different Δ*Cs*. Based on Figure 18, the first block, including the CBCM core, and also the bias current mirror, show a linear response with respect to the variations of Δ*C*. As shown in Figure 16b, and expected from Equation (15), the integration of *f*_CCO_ over *T*_int_ has an almost linear variation versus Δ*C*. Based on the descriptions explained for the sensor performance, it is also expected to obtain a linear response from the practical circuit, in which the counter completes the integration operation in the digital domain.

### 5.3. The Response of the Whole Practical Sensor, Its Nonlinearity, and Its Temperature Dependency 

Based on Figure 17b, the maximum output frequency of the CCO for Δ*C* = 70 fF is less than 600 MHz. The counter is designed for clock pulses with a frequency that is more than this value (about 1 GHz). Using the LFSR structure and the reduced setup time D flip-flop proposed in [79] in the adapted LFSR helps to meet this requirement

**Figure 19:**
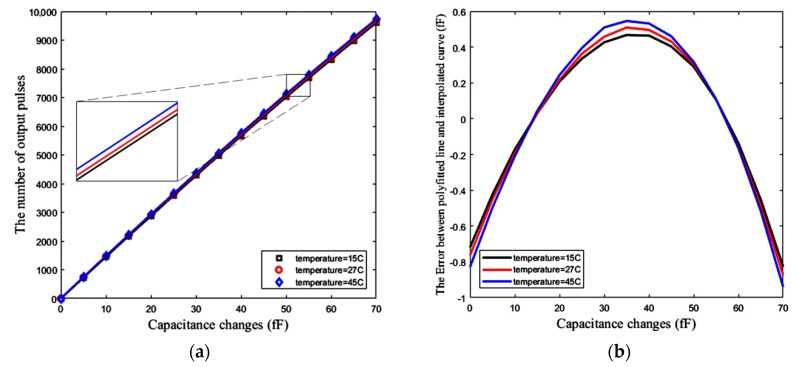
(**a**) Number of pulses versus capacitance changes Δ*C* (fF), (**b**) Error between the simulation results for 15 different values of Δ*C* and the polynomial fitted line (at three different temperatures of 15 °C, 27 °C and 45 °C).

Figure 19a demonstrates the number of pulses that are counted by the counter, versus the changes of the sensing capacitance up to 70 fF. Assuming that the temperature is not changed after calibration, this simulation, along with the associated calibration, is repeated at three different temperatures of 15 °C, 27 °C, and 45 °C. The slope of the polynomial fitted line represents the sensitivity of the sensor, which is about 138 pulses/fF at room temperature (27 °C). Ideally, for a completely linear response, this sensitivity should have a resolution of about 7.2 aF. However, the response is not completely linear. 

One method for the measurement of this nonlinearity is R-squared (R^2^). It is a statistical measure showing how close our response data are to the fitted straight regression line. Based on this method the linearity of the response shown in Figure 19a is approximately R^2^ = 0.9996, meaning that 99.96% of the counter number variations can be explained by a linear model for capacitance changes (Δ*C*) of up to 70 fF. Figure 19b shows the error between the simulated curve obtained from the circuit simulator and the polynomial fitted line (using linear least squares) with this curve for the mentioned range of Δ*C*. Based on this figure, the maximum absolute error due to the nonlinearity of the circuit is about 873 aF, which limits the resolution of the sensor. This nonlinearity also exists in Figure 18b with the same value of R-squared, while Figure 18a has an R-squared value equal to one. This means that the first block, including CBCM core has a good linearity. Thus, the original source of this nonlinearity is the nonlinearity of the CCO, which is predictable by Equations (20) to (24).

In Figure 19a, a higher temperature results in a slight increase in the slope of the fitted line. As shown in Figure 19a,b, the sensor has a negligible temperature dependency.

**Figure 20:**
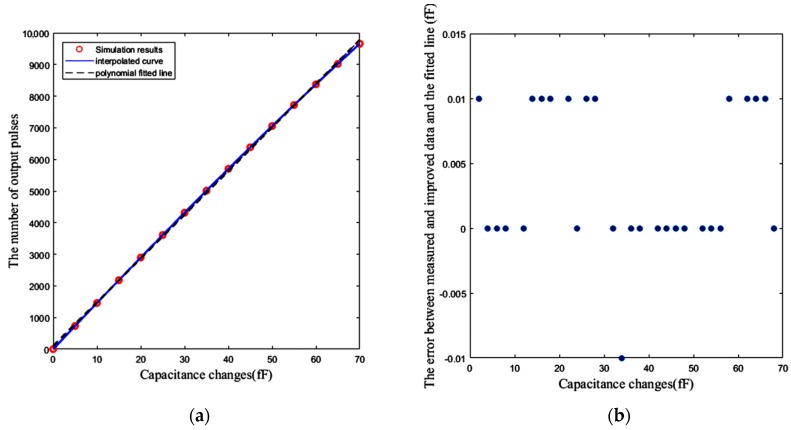
(**a**) Fifteen measured number of pulses versus capacitance changes Δ*C* (fF) at 27 °C, and the interpolated curve and the polynomial fitted line to these 15 points. (**b**) The error between the measured numbers of pulses related to the other values of Δ*C* after pre-distortion, and the polynomial fitted line.

By using intentional pre-distortion, it is possible to achieve better resolution. First of all, the number of output pulses is measured for different values of Δ*C* from zero to 70 fF at 5 fF steps, and a look-up table is formed for these 15 points. This can be done with a capacitor bank. The interpolation of these points gives us an estimation of the trend of the curve for other values of Δ*C*. Thereafter, the interpolated curve can be utilized for pre-distortion in order to reduce the effect of the nonlinearity. Figure 20a shows the number of output pulses for 15 values of known Δ*Cs* in 5 fF steps. Additionally, the polynomial-fitted line and the interpolated curve are depicted in this figure. For any other measured number of pulses, the nearest point of the interpolated curve to the measured value is selected. Based on the interpolated curve, the corresponding Δ*C* can be estimated. As illustrated in Figure 20b, if the difference between the interpolated curve and the polynomial fitted line for the related Δ*C* is subtracted from the measured numbers of pulses, the value of corresponding Δ*C* is estimated by the pre-distorted point and the fitted line with an error less than 10 aF. In other words, interpolation and pre-distortion techniques provide a resolution of about 10 aF for Δ*C*. 

### 5.4. Mismatch Effects

**Figure 21:**
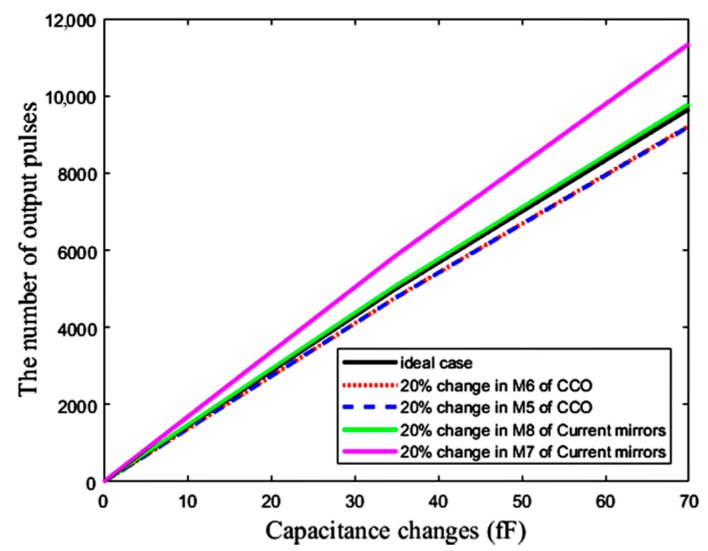
The effect of mismatch error on the capacitive sensor output after the elimination of offset.

The effects of mismatch errors are depicted in Figure 21. A total of 20% of changes in the width of the transistors M_6_ and M_5_ of the CCO (see Figure 10) and M_8_ and M_7_ of the current mirrors (see Figure 4) affect the sensitivity of the CCO and the aspect ratio of the current mirrors, respectively, which both change the sensitivity of the sensor. Mismatch errors cause some offsets in the response of the sensor that can be omitted by the down-counting approach. However, the mismatch of M_7_ of the current mirrors results in a more destructive impact on the response of the sensor, because it changes the aspect ratio *K*_1_ in Equation (6), leading to both the slope and offset variations of the response with respect to Δ*C*. Thus, in spite of the elimination of the offset by the counter, we need a two-point calibration to correct the slope variations, due to mismatch.

### 5.5. Other Specifications of the Sensor

**Figure 22:**
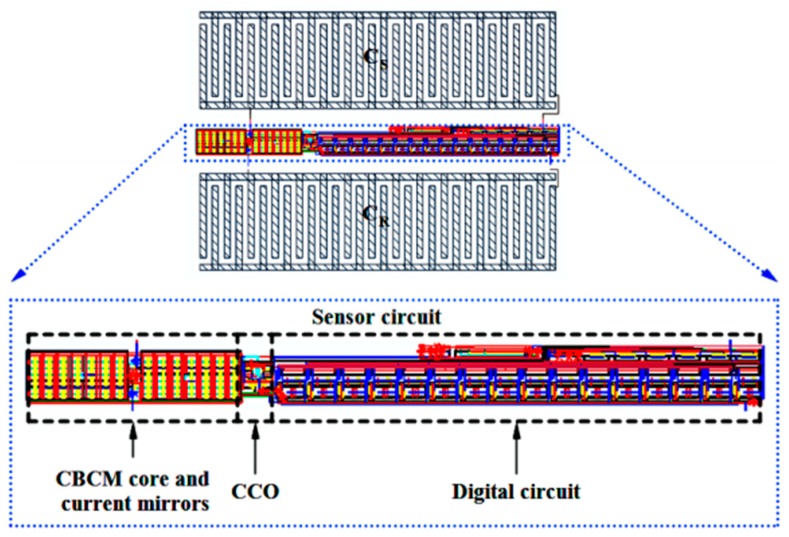
Layout of the proposed capacitive sensor, along with sensing and reference electrodes.

**Figure 23:**
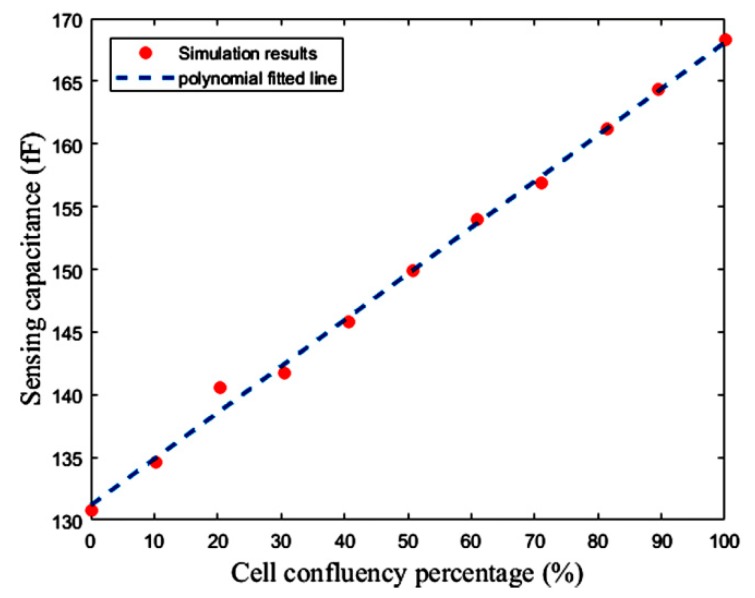
Sensing capacitance versus fibroblast cell confluency percentage.

Figure 22 shows the layout of the proposed capacitive sensor, along with the reference and sensing interdigitated electrodes. The total area occupied by the interface circuit is 24 μm × 300.98 μm, and the total area of one cell of this capacitive sensor is 214.275 μm × 300.98 μm. The interdigitated electrodes are simulated in Sonnet software to estimate the value of reference capacitor and the effect of the cell confluency percentage on the value of the sensing capacitor (*C*_S_). The number of fingers, the spaces between the fingers and the fingers overlaps of the electrodes are 15, 5 μm, and 60 μm, respectively. Thus, the area of one interdigitated electrode is 80 μm × 295 μm, which can be changed based on the application and the accessible chip area. Here, fibroblast cells with an electrical conductivity of 5 S/m and a relative permittivity equal to 1 are used to examine this concept. Figure 23 illustrates the roughly linear upward trend of the value of *C*_S_ for increasing the cell confluency percentage. The characteristics of the proposed sensor are summarized in Table 3. The powers of the two first blocks, including the CBCM core and the CCO, are dynamic, which are reported for 35 fF and a sampling frequency of 100 kHz, in Table 3. 

## 6. Further Discussions and Future Works

The characteristics of the simulated circuit are compared with the other core-CBCM capacitive sensors in Table 4. The simulation results of the proposed circuit indicate that it has a considerably wider IDR, in comparison to the previous works. Here, the IDR and the nonlinearity of the CCO and the size of the counter are the limiting factors for the IDR of Δ*C*. However, working in current-mode assists with having an appropriate IDR. Moreover, the sensitivity of the sensor can be off-chip-controllable, and it tends to vary by the cycles (*N*) of the integration time (*T*_int_ = *NT*_S_): the more quantitative the cycles, the greater the achievable sensitivity. 

**Figure 24:**
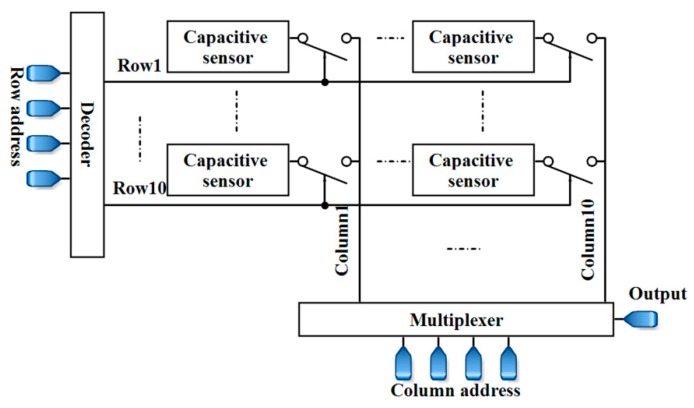
A 10 × 10 array of the proposed capacitive sensor.

To sum up, the results suggest that the proposed capacitive sensor can be a candidate form of architecture for LoC applications such as cellular monitoring. Systematically speaking, the platforms used in these applications require microfluidics to manipulate, control, and steer the aquatic sample towards the electrodes of the biosensor. Packaging microfluidics and CMOS biosensors, and their biocompatibilities are some important issues in these applications. The fabrication of this capacitive sensor, along with microfluidics and a suitable packaging, is considered as future works. Further experiments should be conducted for the evaluation of the sensor for different life science applications. Additionally, controlling the environmental temperature can be an effective factor that should be considered. Furthermore, an array of the sensor-like structure that is shown in Figure 24 makes it possible to perform measurements for different samples simultaneously and paves the way for high-throughput screening. Since the proposed capacitor can register the data, different measurements can be done simultaneously. Then, the registered data can be selected and read by a multiplexer. 

## 7. Conclusions

Previously reported core-CBCM circuits operate based on a current-to voltage-integrator. This paper presents a new core-CBCM capacitive sensor with a wide input dynamic range using a current-mode method, and internal capacitors of CMOS transistors. This new design consists of a high-speed current-controlled oscillator and a fast counter, in order to convert the output current of the CBCM block to variable pulse frequencies. The simulation results indicate a linear relationship between the capacitance and the sensor’s output, with an input capacitance range of around 70 fF, and an absolute error of less than 873 aF. Based on these results, the proposed core-CBCM circuit with an improved IDR and high resolution is the best candidate for the development of high throughput capacitive sensor arrays that are suitable for life science applications. 

## Figures and Tables

**Table 1:** Forward and reverse linear feedback shift register (LFSR) with three different sizes (the rectangles stand for D flip-flops).

**The Length of LFSR**	**Feedback Polynomials for Forward Counting**	**Feedback Polynomials for Reverse Counting**
3 bits	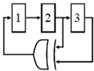 $x^{3}+x^{2}+1$	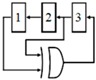 $x^{3}+x^{1}+1$
4 bits	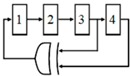 $x^{4}+x^{3}+1$	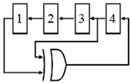 $x^{4}+x^{1}+1$
5 bits	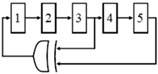 $x^{5}+x^{3}+1$	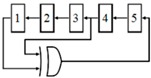 $x^{4}+x^{1}+1$

**Table 2:** Five phases of the sensor and the related situations of the controlling signal.

State	Input of Counter	S_0_ (INC)	S_1_ (CYC)	S_2_ (CNT)	SM0 (of Sel3)	SM1 (of-Sel3)	SM2 (of-Sel3)	S (of-Sel3)	Sb (of-Sel3)
Initialization	*RCLK*	1	0	0	0	1	0	1	0
Down counting (Calibration for Δ*C* = 0)	The output of CCO	0	0	0	0	0	1	0	1
Saving	Ground voltage (*GND*)	0	0	0	1	0	0	0	1
Up counting	The output of CCO	0	0	1	0	0	1	1	0
Cycling	*RCLK*	0	1	0	0	1	0	1	0

**Table 3:** Core-CBCM capacitance-to-frequency converter specifications.

Parameter	Value
Technology	0.18 μm
Supply voltage	1.8 V
Each electrode area	80 μm × 295 μm
Interface circuit area	24 μm × 300.98 μm
Power consumption (for 35 fF)	~ 103 μW
▪ CBCM and current mirrors	4.2 μW
▪ CCO	90.234 μW
▪ Digital circuit	1.3 μW
Sensitivity	138 pulses/fF
Sampling frequency	100 kHz
Dynamic range	70 fF
Linearity (R^2^)	0.9996
Resolution	
▪ In the ideal case without considering the error due to nonlinearity	~8 aF
▪ By considering the maximum error due to nonlinearity	~873 aF
▪ With interpolation and pre-distortion	~10 aF

**Table 4:** Comparison of CMOS capacitive biosensors.

Power	Chip Area (μm^2^)	The Number of Arrays	Voltage Output Type	Capacitance Resolution (aF)	Sensitivity	IDR of Δ*C* (fF)	Supply Voltage (V)	Tech.	Principle	Ref.
29 μW	10^5^	16 × 16	Analog	450 aF	55 mV/fF	0.45 −57	-	0.25 μm	^1^ ChR (^2^ ChS)	[20]
-	-	320 × 320	Analog	21 aF	345 mV/fF	-	-	0.35 μm	ChR (^3^ CSA)	[15]
8 mW	3.6 × 10^5^	4 × 4	Digital	17.5 aF	590 kHz/fF	12 fF	3.3	0.35 μm	^4^ C2F (^5^ RO)	[23]
84 mW	6 × 10^6^		Digital	0.065 aF	32	<1	3.3	0.35 μm	Lock-in	[43]
-	6.272 × 10^5^	4	Analog	10	1 V/fF	2	5	0.8 μm	^6^ CBCM	[61]
-	2 × 10^6^	3	Digital	10	255 mV/fF	~2.7	1.8	0.18 μm	CBCM	[62]
^8^ 3.06 × 10^−3^ μW (core) (at 1 kHz) 1.65 × 10^2^ μW (Buffer amplifier)	^7^ 1.45 × 10^2^	6 × 6	Analog	15	200 mV/fF	25	±3	0.5 μm	CBCM	[64]
580 (at 150 kHz)	10^4^	1	Digital	10	350 mV/fF	10	±3.3	0.35 μm	CBCM	[65]
910 pJ/cycle at (1 kHz)	4.3 × 10^4^	1	Analog	-	23.4 mV/pF	-	-	0.35 μm	CBCM	[74]
1.5 × 10^4^ μW (for 1–70 MHz)	2.5 × 10^12^	256 × 256	Digital	1	-	<1.8	1.2	90 nm	CBCM	[73]
103 μW (for 35 fF at 100 kHz)	6.45 × 10^4^	1	Digital	^9^ 873, ^10^ 10	138 pulses/fF	~70	1.8	0.18 μm	CBCM	This work

^1^ Charge redistribution, ^2^ Charge sharing, ^3^ Charge sensitive amplifier, ^4^ Capacitance-to-frequency converter, ^5^ Ring oscillator, ^6^ Charge-based capacitance measurement. ^7^ This is the area of only one sensor pixel without electrodes and sensor evaluation modules. ^8^ Total power consumption is not reported, ^9^ Without pre-distortion, ^10^ With pre-distortion.
